# Honeybee rebel workers invest less in risky foraging than normal workers

**DOI:** 10.1038/s41598-018-27844-w

**Published:** 2018-06-21

**Authors:** Karolina Kuszewska, Krzysztof Miler, Michal Woyciechowski

**Affiliations:** Institute of Environmental Sciences, Jagiellonian University, Krakow, Poland

## Abstract

In eusocial insect colonies, workers have individual preferences for performing particular tasks. Previous research suggests that these preferences might be associated with worker reproductive potential; however, different studies have yielded inconsistent results. This study constitutes the first comparison of foraging preferences between genetically similar normal and rebel honeybee workers, which present different reproductive potential. We found that rebels, which have a higher reproductive potential than normal workers, displayed a delayed onset of foraging and a stronger tendency to collect nectar compared with normal workers. These results support the hypothesis that workers with high reproductive potential invest more in their own egg laying and avoid risky tasks such as foraging. In contrast, the results do not support the hypothesis that reproductive workers initiate foraging earlier in life than normal workers and specialize in pollen foraging.

## Introduction

Eusocial insect colonies provide examples of extremely complex social behaviours^1,2^. Understanding how the developmental ground plans of solitary ancestors evolved to produce eusocial descendants is one of the most challenging questions in evolutionary biology^3^. It has been suggested that the origin of the worker caste might lie in modifications to the basal reproductive cycle of the solitary ancestral insect. In this model, called the ovarian ground plan hypothesis (OGPH^3^), queens retain some of the characteristics of their solitary ancestors during their reproductive phase, i.e., active ovaries and high titres of vitellogenin, an egg precursor protein^4^, whereas workers resemble the solitary ancestor in its foraging phase, which is characterized by inactive ovaries and low titres of vitellogenin.

In social insects, there is a further division of labour based on the performance of different tasks by different workers. In many species, this non-reproductive division of labour is related to age (temporal) polyethism. Workers typically shift from safe inside-nest tasks to risky outside-nest foraging, and foragers often have preferences for collecting nectar or pollen^5^. Amdam *et al*.^6,7^ developed the OGPH further to obtain the reproductive ground plan hypothesis (RGPH), which states that in workers, genes once in control of reproduction have been rewired to control the division of labour, mainly in terms of foraging behaviours. According to the RGPH, workers with higher numbers of ovarioles in ovaries, a characteristic associated with increased productivity, initiate foraging earlier in life and are more likely to bias foraging towards high-protein types of food (e.g., pollen in bees) than are workers with fewer ovarioles^6,7^.

Experimental support for the RGPH comes primarily from studies of two honeybee lines selected for high and low pollen hoarding^8^. Compared with workers from the low pollen hoarding line and unselected workers, workers in the high pollen hoarding line were shown to have higher reproductive potential, to start foraging earlier in life, to carry larger pollen loads more frequently, and to have elevated levels of vitellogenin^6,9^. Other evidence supporting the RGPH comes from physiological studies focusing on the associations among ovary size, synthesis of the egg yolk protein vitellogenin in the fat body, and foraging specialization. After vitellogenin synthesis knockdown, bees initiate foraging earlier in life and collect larger loads of nectar than workers with normal vitellogenin synthesis^10^. Other studies have shown that bees with a surgically increased ovarian mass begin foraging at an earlier age and specialize in pollen collection^11^.

An alternative hypothesis seeking to explain the relationship between the reproductive potential of workers and their foraging behaviour was described by Schmidt-Hempel^12^. His reproductive conflict and work hypothesis (RCWH) argues that individuals with higher reproductive potential should wait as long as possible for favourable conditions to lay their own eggs and therefore should avoid risky foraging in general compared with individuals with lower reproductive potential. The experimental support of predictions derived from this hypothesis comes from studies on different species of social insects, including bumble bees^13^, ants^14^, and wasps^15^, along with ‘anarchistic’ bees (*Apis mellifera* strains selected for high rates of worker reproduction)^16^, the Cape honeybee (*A*. *m*. *capensis*)^17^ and the Asian hive bee (*A*. *cerana*)^18^. These studies show that individuals with higher productivity typically avoid foraging and display no foraging preference when engaged in this task.

Studies with conflicting results have led to active discussion regarding the evolution of division of labour. Some researchers suggest that the confusion concerning this issue stems from unreliable results obtained from artificially selected lines of bees, after genetic manipulation (i.e., with knockdown genes) or artificial transplantation of organs, or deduced from comparisons between different bee species^10,16,18^. An alternative approach for comparing the behaviour and reproductive biology of genetically distinct individuals is to compare the behaviours of reproductive and non-reproductive individuals in a standardized genetic background. Here, we performed the first comparison of the foraging characteristics of normal and rebel workers of the honeybee *A*. *mellifera*. These two groups of workers comprise genetically similar sisters that differ in their reproductive potential. Compared with normal workers, rebel workers are more engaged in laying their own male-determined eggs than in rearing the queen’s offspring^19^. Therefore, they exhibit significantly more ovarioles in their ovaries and more developed mandibular glands, as observed in a queen, in addition to exhibiting underdeveloped hypopharyngeal glands (HPGs), which suggests a low production of brood food^20,21^. Differences between rebel and normal workers are based solely on the environmental conditions experienced during the larval period; specifically, rebels develop in the absence of a queen or, more precisely, in the absence of a queen’s mandibular gland pheromone^22^. The situation in which normal and rebel workers develop together in a colony can be easily arranged experimentally using artificially split colonies that mimic a temporary lack of a queen at swarming^20,23^. In the present study, we tested two alternative hypotheses with conflicting predictions: if the RGPH better explains the relationship between worker reproductive potential and foraging behaviour, we expect that rebel workers will begin foraging earlier in life and preferentially collect pollen over nectar; however, if the RCWH better explains this relationship, we expect that rebel workers will initiate foraging later in life than normal workers and will either preferentially collect nectar over pollen or exhibit no foraging preference.

Similar to previous research^20,21^, we show that workers that develop in queenless conditions at the larval stage have more ovarioles in their ovaries (Supplementary Fig. S1a) and smaller HPGs (Supplementary Fig. S1b) than workers reared in queenright conditions. The former develop into rebel workers, and the latter develop into normal workers. Interestingly, we also observed that a higher number of filaments in the ovary was associated with ovariole swelling (Supplementary Fig. S2), a finding reported previously in a Brazilian population of *A*. *mellifera*^24^, anarchistic bees^16^, and bees selected for low and high pollen hoarding^6^ (see also^17,18,25^ for other results). This result supports the view that rebel workers not only have a higher reproductive potential than normal workers but are also more engaged in laying their own male-determined eggs than in rearing the queen’s offspring in their adult life^20^.

**Figure 1:**
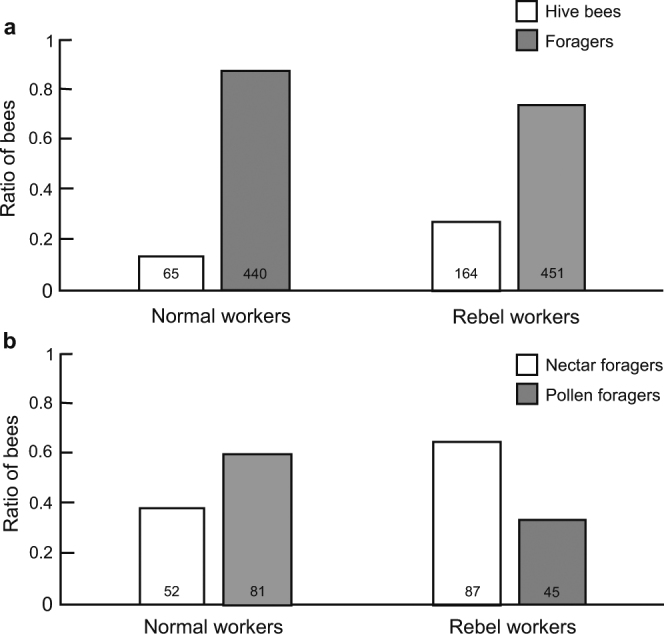
Ratios of honeybee workers performing different tasks. (**a**) Ratio of in-hive and foraging workers on the 25^th^ day of life (Fisher’s exact test, *P* < 0.0001). (**b**) Ratio of workers collecting nectar or pollen (Fisher’s exact test, *P* < 0.0001). The number at the bottom of each bar indicates the sample size.

**Figure 2:**
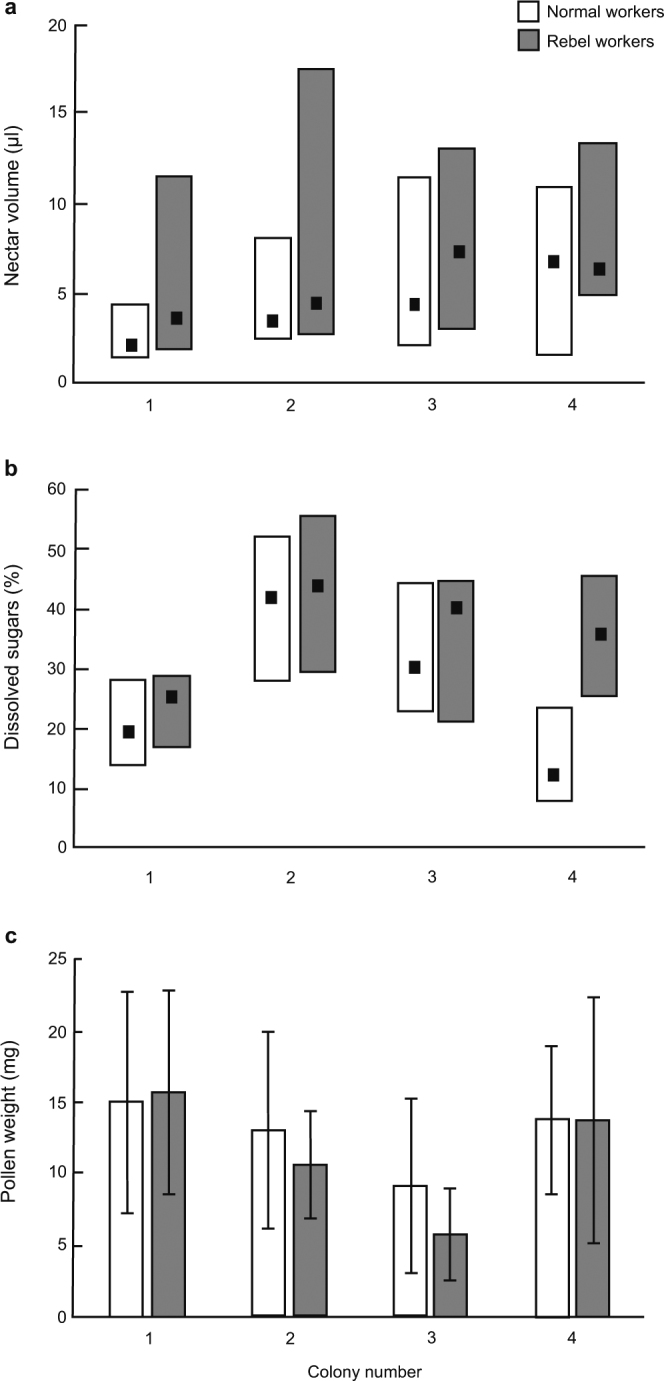
Loads carried by honeybee workers. (**a)** Nectar/crop volume collected by workers (medians and quartiles; GLZ, Wald’s χ^2^ = 48.01, *P* < 0.001). (**b**) Sugar concentration of nectar (medians and quartiles; GLZ, Wald’s χ^2^ = 54.58, *P* < 0.001). (**c**) Weight of pollen collected (means ± SDs; two-way ANOVA, F_1,3_ = 2.00, *P* = 0.204).

**Figure 3:**
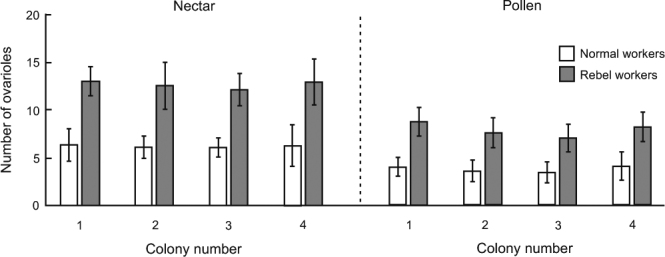
Number of ovarioles (mean ± SD) in honeybee workers foraging for nectar and/or pollen. (Three-way ANOVA, F_3,257_ = 140.17, *P* < 0.001; post hoc Tukey test, nectar normal workers *vs*. nectar rebel workers *P* < 0.001, nectar normal worker *vs*. pollen normal workers *P* < 0.001, nectar rebel workers *vs*. pollen rebel workers *P* < 0.001, nectar rebel workers *vs*. pollen normal workers *P* < 0.001).

To determine whether the RGPH or the RCWH better explains the relationship between the reproductive potential of workers and their non-reproductive division of labour, the onset of foraging and the foraging preferences of workers were analysed. We found that rebel workers avoid foraging; fewer of these workers started foraging from the 25^th^ day of life (73%) compared with normal workers (87%; Fig. 1a). This result is in accordance with the expectations of the RCWH^12^ and with previous results obtained using *A*. *m*. *capensis*^17^ and anarchistic bees^16^ as model organisms. Furthermore, rebel workers foraged more often for nectar (66%) than for pollen (34%), whereas normal workers foraged more often for pollen (61%) than for nectar (39%; Fig. 1b). In addition, the nectar collected by rebel workers was of higher volume (Fig. 2a) and was more concentrated (Fig. 2b) than the nectar collected by normal workers, although both rebel and normal workers that foraged for pollen brought similar pollen loads to the hive (Fig. 2c). Moreover, a higher number of ovarioles was associated with a preference for nectar collection in both rebel and normal workers (Fig. 3). A foraging preference for nectar in workers that are characterized by higher reproductive potential, as in the case of rebel workers, is also consistent with the RCWH because this task entails lower risks than foraging for pollen. Each foraging trip is energetically costly, and the foraging bee can find itself out of fuel during foraging^26^. Bees foraging for nectar can use their collection stored in their crop on their way back to the hive, whereas bees foraging for pollen are not guaranteed this option because plants used as pollen sources do not necessarily offer any nectar^27^.

Our results do not support the RGPH as an explanation for preferences in the non-reproductive division of labour. The central point of this hypothesis is that the number of ovarioles and the signals the ovary mediates via vitellogenin synthesis in the fat body regulate foraging preference^6,7,10^. We did find a positive association between ovary size and ovary activation, which is consistent with the RGPH; however, we did not find that bees of higher reproductive potential initiate foraging at an earlier age than bees of lower potential or that they prefer foraging for pollen. In contrast, the predictions of the RCWH were upheld, which suggests that the evolution of a non-reproductive division of labour in insect societies emerges from variation in the reproductive conflict among colony members^12^. The avoidance of risky tasks by individuals of higher reproductive potential is also in accordance with the life-history and kin selection theories. The workers of social insects face a trade-off between investing energy in their own reproduction or in helping with colony maintenance, similarly to how solitary animals face a trade-off between the current and future allocations of energy for reproduction^15^. Therefore, if some workers have a predisposition for future reproduction, they avoid investing energy in performing tasks, particularly risky ones, in the nest and instead wait for the opportunity to lay their own eggs.

In summary, the foraging preferences of honeybee workers are strongly associated with their reproductive traits, and these results can likely be generalized to other social insects. However, preferences in foraging behaviour do not appear to rely on the modifications of existing gene networks alone, as proposed by the RGPH. Our novel results clearly suggest that the major factor in shaping worker foraging preferences is reproductive conflict among individuals. The findings increase our understanding of the origins of the division of labour in insect societies.

## Methods

The study was performed in May and June 2015. Four unrelated queenright honeybee (*A*. *m*. *carnica*) colonies, each consisting of 20 000–40 000 workers and headed by naturally mated queens, were studied. Initially, rebel and normal workers were reared in all colonies (see Supplementary Methods) as described by Woyciechowski and Kuszewska^20^. After their development, workers (two groups, rebel and normal) were allowed to emerge within 24 h in the incubator. These workers from all colonies and groups were colour-marked on their thorax with a spot of paint and then returned to their native hives.

Starting 10 days later, the entrance counts of marked bees returning from foraging trips (recorded over 10 min at the foraging period peak of 11:00 am to 12:00 pm) were noted for several days; when the counts levelled off, the collection of marked workers was initiated. When the counts levelled off (day 46: in colonies 1 and 2, workers were 24 days of age; whereas in colonies 3 and 4, workers were 25 days of age), a set of workers was collected. These workers were used to determine the foraging preferences for nectar or pollen collection in rebel and normal workers. Foragers from both groups were collected between 9:00 am and 1:00 pm. The number of collected workers depended on the colony and treatment (numbers of bees: colony 1: 40 non-rebel and 40 rebel workers; colony 2: 40 non-rebel and 40 rebel workers; colony 3: 23 non-rebel and 23 rebel workers; colony 4: 40 non-rebel and 40 rebel workers). For each bee, (1) the mass of carried pollen (if present), (2) the volume of nectar in her crop, and (3) the concentration of sugar in the crop solution were determined. All of the collected workers were frozen (−40 °C) for the subsequent dissection of ovarioles and HPGs.

When the marked workers were 25 days old, the two experimental groups of workers (normal vs. rebel workers) were assessed to determine which had the higher ratio of foraging bees. Native colonies of experimental bees were moved several metres from their original site, and new, empty hives (without bees) with wax frames were placed at the original sites. As a result, only nurse bees remained in the native colonies (in new locations), whereas all foragers returning from the field went into the new empty hives (in the old locations)^28,29^. On the evening of the same day, all marked foragers and bees working inside the nest were counted. The number of foraging bees for each colony was estimated from the sum of workers collected during the determination of the foraging preferences of workers and marked foragers collected from the new, empty hives. The number of non-foraging workers for each colony was estimated by counting the marked workers that were present and remained in the old nests in the new locations.

### Examination of ovaries and hypopharyngeal glands

The ovarioles and HPGs of the frozen workers were dissected and examined under a stereomicroscope. The number of ovarioles summed over both ovaries was noted, and ovary development was assessed. To evaluate ovary development, the most developed ovariole of each ovary was selected, and the maximum diameters of these two ovarioles (maximum width) were measured following Nakaoka *et al*.^30^, according to whom the ovariole diameter accurately reflects ovarian activity. The size of the hypopharyngeal glands was calculated from the average of 10 acini (square root of longest × shortest diameters of five acini from the right gland and five from the left gland). The HPG consists of a great number of lobes, called acini, and their diameter is routinely used as an index of gland size^20,31^. All organs were stained with Giemsa reagent for approximately 10 seconds before measurement.

### Statistical analysis

To compare anatomical parameters (number of ovarioles, size of hypopharyngeal glands) and the masses of collected pollen between rebel and normal workers, two-way ANOVA was used. The association between ovariole number and size was tested through simple regression separately in normal and rebel workers. Differences in the volume of collected nectar and sugar concentration in the crops between rebel and normal foragers were analysed using generalized linear model/nonlinear models (GLZ) specifying a Poisson distribution and the log link function, which is a semiparametric statistical test^28^.

In a foraging preference experiment, the numbers of foragers and workers that remained in the nest (non-foragers) and the numbers of pollen and nectar foragers between rebel and normal workers were compared using Fisher’s exact tests. To determine whether the normal and rebel workers that preferred foraging for nectar were anatomically different (number of ovarioles, size of hypopharyngeal glands) from individuals who preferred foraging for pollen, a mixed model three-way ANOVA was used.

## Electronic supplementary material

Supplementary Information
