# Supplementary material for: Honeybee rebel workers invest less in risky foraging than normal workers
Source: Sci Rep. 2018 Jun 21;8:9459. doi: 10.1038/s41598-018-27844-w (PMC6013497; doi:10.1038/s41598-018-27844-w)
Supplement: Supplementary file 1 — Supplementary Information [file 41598_2018_27844_MOESM1_ESM.pdf]

Supplementary Information

Honeybee rebel workers invest less in risky foraging than normal workers

Karolina Kuszewska[1*], Krzysztof Miler[1], Michal Woyciechowski[1]

Supplementary Figures

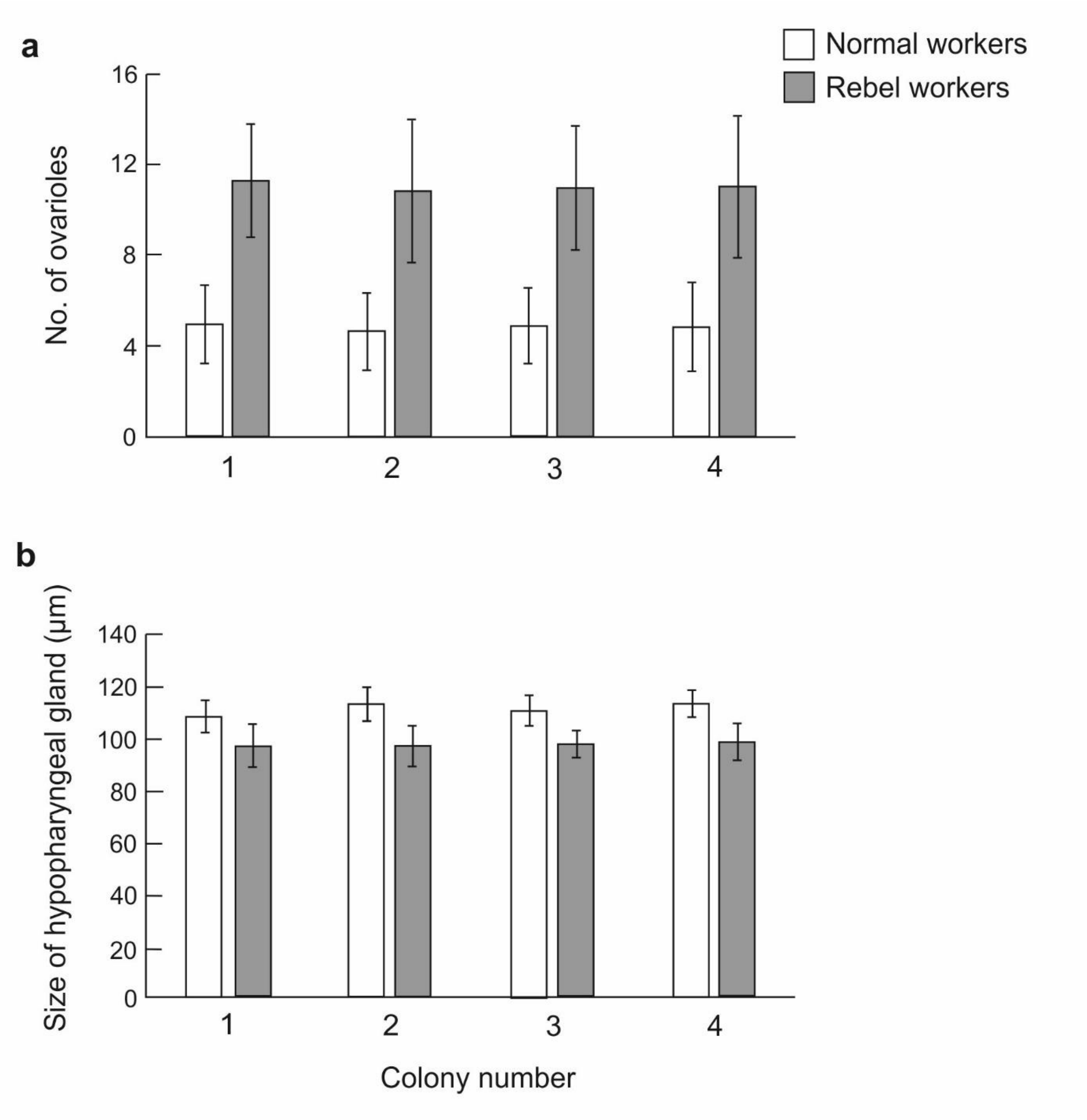

Figure S1 | Anatomical parameters of honeybee workers reared under different conditions during the larval stage. a, Number of ovarioles (means ± SD, two-way ANOVA, $F_{1,3}$ = 6552.02, $P < 0.001$). **b,** Size of hypopharyngeal gland (means ± SD, two-way ANOVA, $F_{1,3}$ = 143.45, $P = 0.001$).

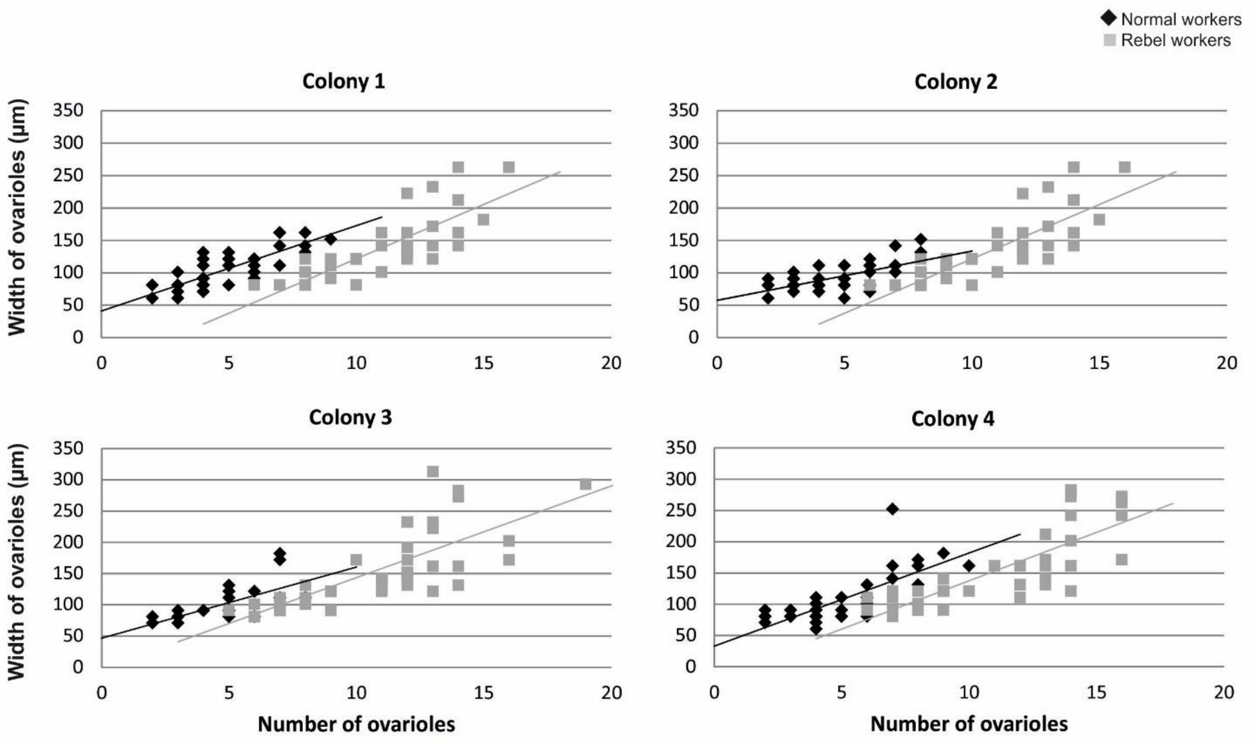

Figure S2 | Relationship between ovariole number and development in bees obtained from the four tested colonies. (Simple regression, Colony 1: normal workers, β = 0.804, R^2 = 0.638, $F_{1,38}$ = 69.691, P < 0.001; rebel workers, β = 0.805, R^2 = 0.638, $F_{1,38}$ = 69.803, P < 0.001; Colony 2: normal workers, β = 0.626, R^2 = 0.375, $F_{1,38}$ = 24.450, P < 0.001; rebel workers, β = 0.754, R^2 = 0.557, $F_{1,38}$ = 50.131, P < 0.001; Colony 3: normal workers, β = 0.640, R^2 = 0.381, $F_{1,21}$ = 14.552, P = 0.001; rebel workers, β = 0.593, R^2 = 0.321, $F_{1,21}$ = 11.411, P = 0.003; Colony 4: normal workers, β = 0.763, R^2 = 0.571, $F_{1,38}$ = 52.965, P < 0.001; rebel workers, β = 0.796, R^2 = 0.623, $F_{1,21}$ = 65.608, P < 0.001).

Supplementary methods

Bee Rearing

The research was conducted in May and June 2015 in the experimental apiary of the Institute of Environmental Sciences (Jagiellonian University, Krakow, southern Poland). Four queenright honeybee (*A. m. carnica*) colonies, each consisting of 20 000-40 000 workers and headed by naturally mated queens, were studied. All of the colonies were treated the same way, and the procedures were started on four successive days for each of the colonies. The experiment started when the queen was confined to two experimental frames to produce eggs of a similar age (day 0). Three days later, each colony was divided into queenright and queenless subunits (equal distributions of resources, honeybee broods and numbers of returning foragers), each with one experimental frame (day 3). When the worker cells on the experimental frames were sealed (day 12), the subunits were reunited. As a result, the experimental broods were maintained under the same conditions during their prepupal and pupal stages. Twenty-two days after the start of the experiment, the frames with newly emerged workers reared as larvae under queenright and queenless conditions were inserted into an incubator in the laboratory (34°C, 90% RH). All workers that emerged within 24 h were used in the experiment. The workers from all colonies and groups were marked on the thorax with a spot of paint (Marabue-Brilliant Painter), and all were returned to their native hives to conduct the sucrose sensitivity and foraging preference experiments at a later time.

Foraging Preference

The entrance counts of marked bees returning from foraging trips (recorded for 10 min during the peak foraging period: 11:00 am to 12:00 pm) were logged beginning on day 32 of the

experiment (when the workers were 10 days of age). When the counts plateaued (day 46, when the workers were 24 and 25 days of age), a set of workers was collected. These workers were used to determine the foraging preference for nectar or pollen of rebel and non-rebel workers. The foragers from both groups were collected between 9:00 am and 01:00 pm. The number of collected workers depended on the colony and treatment (numbers of bees: colony 1: 40 non-rebel and 40 rebel workers; colony 2: 40 non-rebel and 40 rebel workers; colony 3: 23 non-rebel and 23 rebel workers; colony 4: 40 non-rebel and 40 rebel workers). For each bee, the following parameters were determined: (1) the mass of pollen she carried (if present), (2) the volume of nectar she carried in her crop, and (3) the concentration of sugar in the solution carried (if present). All of the collected workers were frozen (-40°C) for subsequent dissection of ovarioles and hypopharyngeal glands (HPGs).

The pollen loads were removed from two corbiculae for each bee and weighed to determine the mass of the pollen loads. To retrieve nectar from the crop, the abdomen of each captured bee was gently squeezed between the thumb and forefinger to cause regurgitation of the crop contents [1]. These contents were drawn into a 10-µl microcapillary tube, and the length of the liquid column was measured with a ruler and then converted into volume in microliters [1,2]. The percentage of dissolved sugars in the regurgitated crop was subsequently measured using a handheld refractometer (hand refractometer, Atago HSR-500) with a range of 0-80 brix. This enabled estimation of the sugar content brought by foragers to the hive.

Ratio of Foraging Bees

The day after the preference foraging experiment (day 47 of the experiment, when the workers were 25 days of age), the two experimental groups of workers (reared under queenright and queenless conditions) were assessed to determine which had a higher ratio of foraging bees.

To do so, the native colonies were moved several metres from the original colony site, and new empty boxes (without bees) with wax frames were placed in the original locations of the native colonies, with their entrances facing the same direction as those of the native hives. As a result of this manipulation, only nurse bees remained in the native colonies (in the new locations) because all of the foragers in the field returned to the new empty colonies (in the original locations) after leaving their native colonies (according to the method described by Kuszewska & Woyciechowski[3]; similar methods were used in a study described by Amdam et al.[4] and reviewed by Hammer & Menzel[5]). On the evening of the same day, all marked foragers and bees working inside the nest (from both rebel and non-rebel worker groups) were counted. The numbers of foraging bees were estimated based on the total numbers of workers collected during the foraging preference experiment (24-day-old foragers) and the bees collected from the new hive boxes that remained in the original locations. The numbers of non-foraging workers were estimated by counting the workers that remained in the old nests in the new locations.

Examination of Ovaries and Hypopharyngeal Glands

The ovarioles and hypopharyngeal glands of frozen workers were dissected and examined under a stereomicroscope. The ovarioles in both ovaries (total) were counted, and an assessment of ovary development was then performed. To assess ovary development, the most developed ovariole of each of the ovaries was selected, and the maximum diameters of the two ovarioles (maximum width) were measured as described by Nakaoka et al.[6], who reported that the ovariole diameter accurately reflects ovarian activity. The size of each hypopharyngeal gland was calculated from the average of 10 acini (square root of longest × shortest diameters of five acini from the right gland and five from the left gland [3,7,8]. The hypopharyngeal gland consists of a large number of lobes, called acini, and their diameter is routinely used as an index of gland

size [6,9]. All organs were stained with Giemsa reagent (for approximately 10 seconds) before measurement.

Statistical Analysis

To compare anatomical parameters (number of ovarioles and size of HPGs) as well as the mass of collected pollen between rebel and normal workers, a mixed model two-way ANOVA was used, with the type of worker (rebel *vs*. normal) as a fixed effect and colony as a random effect. If an experimental comparison was statistically significant, the ANOVA was followed by multiple comparisons using the post-hoc Tukey HSD test, with $P < 0.05$ considered significant. The association between ovariole number and size was tested using simple regression separately in normal and rebel workers.

Differences in the volume of collected nectar and sugar concentration in nectar between rebel and normal foragers were analysed using generalized linear model/nonlinear models (GLZ) specifying the Poisson distribution and the log link function, which is a semiparametric statistical test[3,10]. The colony was considered a random effect, and the type of worker (normal *vs.* rebel) was considered a fixed effect.

In the foraging preference experiment, the numbers of foragers and workers that remained in the nest (non-foragers) and the numbers of pollen and nectar foragers between normal and rebel workers were compared using Fisher's exact test. First, we tested whether there were differences among the colonies. There were no differences among the colonies in either the onset of foraging or the foraging preference (see Supplementary Tables S1 and S2); therefore, the data from the colonies were pooled for further analyses using Fisher's exact test.

To determine whether normal and rebel workers that preferred foraging for nectar showed anatomical differences (number of ovarioles and size of HPGs) from those that preferred

foraging for pollen, three-way ANOVA was used, with the type of worker (rebel *vs*. normal) and foraging preference (nectar *vs*. pollen) as fixed effects and colony as a random effect. If an experimental comparison was statistically significant, the ANOVA was followed by multiple comparisons using the post-hoc Tukey HSD test, with $P = 0.05$ considered significant. All of the calculations and analyses for all experiments were performed with STATISTICA 9.0.

1. Oldroyd, B. P. & Beekman, M. Effects of selection for honey bee worker reproduction on foraging traits. *PLoS Biol.* **6,** e56 (2008).
2. Nelson, C. M., Ihle, K. E., Fondrk, M. K., Page, R. E. & Amdam, G. V. The gene vitellogenin has multiple coordinating effects on social organization. *PLoS Biol.* **5,** e62 (2007).
3. Kuszewska, K. & Woyciechowski, M. Reversion in honeybee, *Apis mellifera*, workers with different life expectancies. *Anim. Behav.* **85,** 247–253 (2013).
4. Amdam, G. V *et al.* Social reversal of immunosenescence in honey bee workers. *Exp. Gerontol.* **40,** 939–47 (2005).
5. Hammer, M. & Menzel, R. Learning and memory in the honeybee. *J. Neurosci.* 1617–1630 (1995).
6. Nakaoka, T., Takeuchi, H. & Kubo, T. Laying workers in queenless honeybee (*Apis mellifera* L.) colonies have physiological states similar to that of nurse bees but opposite that of foragers. *J. Insect Physiol.* **54,** 806–812 (2008).
7. Woyciechowski, M. & Kuszewska, K. Swarming generates rebel workers in honeybees. *Curr. Biol.* **22,** 707–711 (2012).
8. Kuszewska, K. & Woyciechowski, M. Age at which larvae are orphaned determines their development into typical or rebel workers in the honeybee (*Apis mellifera* L.). *PLoS One*

10, e0123404 (2015).

9. Wegener, J., Huang, Z. Y., Lorenz, M. W. & Bienefeld, K. Regulation of hypopharyngeal gland activity and oogenesis in honey bee (*Apis mellifera*) workers. *J. Insect Physiol.* **55,** 716–725 (2009).

10. Härdle, W., Mammen, E. & Müller, M. Testing parametric versus semi- parametric modelling in Generalized Linear Models. *J. Am. Stat. Assoc.* **93,** 1461–1474 (1996).

Supplementary Table S1 | Numbers of hive workers and foragers among 24-day-old rebel and normal workers. The results of Fisher's exact tests indicate whether there are differences between normal and rebel workers in foraging behaviour and whether there are differences between bees coming from different colonies.

<table>
<tr><th></th><th colspan="2">Normal workers</th><th colspan="2">Rebel workers</th><th rowspan="3">Fisher's exact test for each colony – P value</th></tr>
<tr><th></th><th colspan="4">Number of workers</th></tr>
<tr><th></th><th>Hive workers</th><th>Foragers</th><th>Hive workers</th><th>Foragers</th></tr>
<tr><td>Colony 1</td><td>16</td><td>141</td><td>63</td><td>168</td><td>0.0001</td></tr>
<tr><td>Colony 2</td><td>25</td><td>175</td><td>21</td><td>120</td><td>0.0550</td></tr>
<tr><td>Colony 3</td><td>4</td><td>35</td><td>18</td><td>35</td><td>0.0124</td></tr>
<tr><td>Colony 4</td><td>20</td><td>89</td><td>52</td><td>128</td><td>0.0499</td></tr>
<tr><td>Fisher's exact test for different colonies – P value</td>
<td colspan="2">
<table>
<tr><th></th><th>Colony 1</th><th>Colony 2</th><th>Colony 3</th><th>Colony 4</th></tr>
<tr><td>Colony 1</td><td></td><td>0.6163</td><td>1.0000</td><td>0.5823</td></tr>
<tr><td>Colony 2</td><td>0.6163</td><td></td><td>1.0000</td><td>0.0582</td></tr>
<tr><td>Colony 3</td><td>1.0000</td><td>1.0000</td><td></td><td>0.6137</td></tr>
<tr><td>Colony 4</td><td>0.5823</td><td>0.0582</td><td>0.6137</td><td></td></tr>
</table>
</td>
<td colspan="2">
<table>
<tr><th></th><th>Colony 1</th><th>Colony 2</th><th>Colony 3</th><th>Colony 4</th></tr>
<tr><td>Colony 1</td><td></td><td>0.3306</td><td>0.0891</td><td>0.7405</td></tr>
<tr><td>Colony 2</td><td>0.3306</td><td></td><td>0.0615</td><td>0.0978</td></tr>
<tr><td>Colony 3</td><td>0.0891</td><td>0.0615</td><td></td><td>0.4982</td></tr>
<tr><td>Colony 4</td><td>0.7405</td><td>0.0978</td><td>0.4982</td><td></td></tr>
</table>
</td>
<td></td></tr>
</table>

Supplementary Table S2 | Numbers of pollen and nectar foragers in 24-day-old rebel and normal workers. The results of Fisher's exact tests indicate whether there are differences between normal and rebel workers in foraging preferences and whether there are differences between bees coming from different colonies.

	Normal workers		Rebel workers		Fisher's exact test for each colony – *P* value
	Number of workers				
	Pollen foragers	Nectar foragers	Pollen foragers	Nectar foragers	
**Colony 1**	23	12	13	23	0.0178
**Colony 2**	22	16	13	25	0.0649
**Colony 3**	10	12	3	18	0.0452
**Colony 4**	26	12	15	22	0.0207

Fisher's exact test for different colonies – *P* value

	**Colony 1**	**Colony 2**	**Colony 3**	**Colony 4**
**Colony 1**		0.6306	0.1719	1.0000
**Colony 2**	0.6306		0.4254	0.4760
**Colony 3**	0.1719	0.4254		0.1043
**Colony 4**	1.0000	0.4760	0.1043	

	**Colony 1**	**Colony 2**	**Colony 3**	**Colony 4**
**Colony 1**		1.000	0.2353	0.8108
**Colony 2**	1.000		0.2486	0.6371
**Colony 3**	0.2353	0.2486		0.1456
**Colony 4**	0.8108	0.6371	0.1456	
